# The Regulatory Mechanism of MLT/MT1 Signaling on the Growth of Antler Mesenchymal Cells

**DOI:** 10.3390/molecules22101793

**Published:** 2017-10-23

**Authors:** Feifei Yang, Changjiu He, Xuyang Sun, Jing Wang, Can Luo, Guoshi Liu, Liguo Yang, Jiajun Xiong, Lijun Huo

**Affiliations:** 1 Key Lab of Agricultural Animal Genetics, Breeding and Reproduction of Ministry of Education, College of Animal Science and Technology, Huazhong Agricultural University, Wuhan 430070, China; yangfeifei20036@163.com (F.Y.); chungjoe@mail.hzau.edu.cn (C.H.); sunxuyangabc@yahoo.com (X.S.); lc7811017@163.com (C.L.); yangliguo2006@foxmail.com (L.Y.); 2 Department of Animal Husbandry and Veterinary, Wuhan Agricultural School, Wuhan 430043, China; 3 National Engineering Laboratory for Animal Breeding, Key Laboratory of Animal Genetics and Breeding of the Ministry of Agriculture, Beijing Key Laboratory for Animal Genetic Improvement, College of Animal Science and Technology, China Agricultural University, Beijing 100193, China; caylajingjing@gmail.com (J.W.); gshliu@cau.edu.cn (G.L.)

**Keywords:** sika deer, melatonin, MT1, mesenchymal cells, IGF1

## Abstract

Melatonin (MLT) plays an important role in regulating the physiological cycle of seasonal breeding animals. Melatonin receptor I (MT1) is effectively expressed in the cambium layer of deer antler. However, the function and metabolic mechanism of MLT/MT1 signaling in the mesenchymal cells of sika deer remain to be further elucidated. In this work, we detected the effects of MLT/MT1 signaling on mesenchymal cells proliferation and the interaction between MLT/MT1 and IGF1/IGF1-R signaling. The results show that (1) deer antler mesenchymal cells actually express MT1; (2) exogenous melatonin significantly promotes mesenchymal cells proliferation, while MT1 knock-down significantly impairs the positive effects of melatonin; and (3) melatonin significantly enhanced IGF1/IGF1-R signaling, as both the expression of IGF1 and IGF-1R increased, while MT1 knock-down significantly decreased IGF1-R expression and IGF1 synthesis. In summary, these data verified that MLT/MT1 signaling plays a crucial role in antler mesenchymal proliferation, which may be mediated by IGF1/IGF1-R.

## 1. Introduction

Antler is an ideal model for organ regeneration research, as it can regenerate periodically after amputation [1,2,3]. During deer antler growth, the periosteum is transformed into mesenchymal tissue, and mesenchymal cells develop into cartilage cells. Mesenchymal proliferation determines the speed of antler growth [4,5]. Mesenchymal cells are considered to be a major cell source of velvet growth, as it can differentiate into fibroblasts, chondrocytes, and osteocytes during velvet growth [6,7]. It was verified that growth factors play a major role in antler development. Insulin-like growth factor I (IGF1) is the crucial growth factor that stimulates antler growth. During velvet regeneration and development, plasma levels of IGF1 are significantly elevated during the velvet antler growth phase, relative to the other phases of pedicle, as well as first antler development, and a strong positive correlation exists between antler growth rate and circulating concentrations of IGF1 [8]. The effects of IGF1 on the growth of the fibroblast and cartilage zones were investigated; IGF1 was found to be mitogenic for cells originating from the perichondrium, mesenchyme, and cartilage zone of antler [9,10]. The localization of IGF1 in chondrocytes and osteoblasts also suggests that IGF1 may play an important role in cartilage and bone formation [11,12].

Melatonin (MLT), a multi-function hormone, plays an important role in the regulation of redox equilibrium, circadian rhythm, sleep, metabolic activities, mood disorders, as well as immunity [13,14,15,16,17,18,19]. It was well-documented that melatonin significantly suppresses experimental carcinogenesis in vivo [20]. Studies have shown that melatonin promotes cell survival and inhibits apoptosis, mainly through its antioxidizing action [21]. The mitochondria are the main source of intracellular free-radicals, and a recent study showed that the mitochondria possess the ability to synthesize melatonin and it was further verified that de novo melatonin production in the mitochondria provides one-site protection, which contributes to mitochondria function and less free-radical production [22]. In addition to its direct antioxidation, the receptor pathway of melatonin also plays an important role in cell proliferation and differentiation. MT1 and MT2, the trans-membrane receptors of melatonin, were studied deeply over the last decade. Recently, it was verified that melatonin promotes granulosa cell luteinization and embryo implantation, which are mediated by MT1 and MT2, respectively [23,24]. Moreover, agomelatine, the newer antidepressant which, acting synergistically on MT1 and MT2 receptors, displays robust anxiolytic-like actions [25,26]. Studies have shown that melatonin promotes antler growth. Oral administration of melatonin shortens the antler cycle of white-tailed deer; the number of cycles per year increased from one to two. Melatonin treatment also induced stag estrus performance and then stimulated antler growth [27,28,29]. Suttie et al. reported that IGF1 levels during the antler cycle are affected by melatonin treatment, and melatonin implantation advanced the seasonal patterns of increases and decreases of plasma IGF1 and also of weight gain and loss. The cessation of melatonin treatment in February produced early antler castings, as well as a second (out-of-season) antler, and increased IGF1 [30]. Our previous works have also verified that antler yield was affected by base mutation of IGF and MT1 receptors [31,32]. To sum up, melatonin participates in the regulation of antler regeneration; however, the mechanisms have not yet been clearly investigated.

In the current study, we conducted a series of experiments to determine whether MLT/MT1 signaling influences the growth of antler mesenchymal cells. Initially, we examined the distribution of melatonin receptors, MT1, and evaluated the effects of exogenous melatonin on cell proliferation. Thereafter, RNAi was adopted to decrease MT1 levels in order to inhibit MLT/MT1 signaling, and cell proliferation as well as cell cycle-related genes (*PCNA*, *CyclinD2*, *p21*, *p27*) were detected. Finally, the effects of MLT/MT1 signaling on IGF1/IGF-1R signaling were also explored.

## 2. Results

### 2.1. Effect of Melatonin on Cell Proliferation

**Figure 1:**
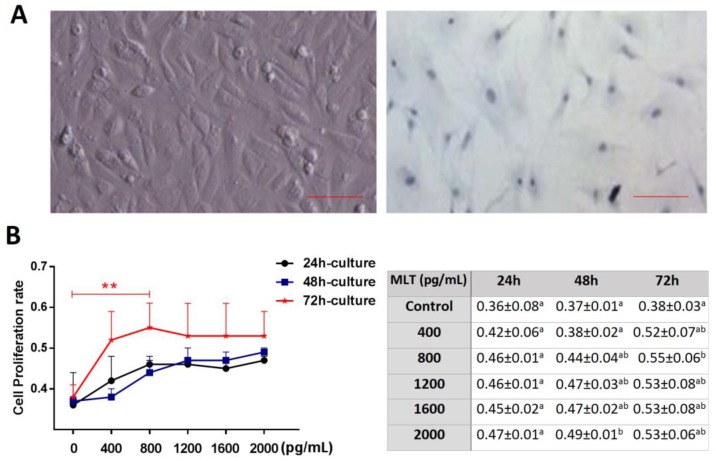
Effects of melatonin on cell proliferation. (**A**) Cell identification using toluidine blue-staining, Scale bar = 100 μm; (**B**) cell proliferation detection using MTT. ** represents significant differences, *p* < 0.01. The different superscript letters (a–b) represent significant differences of these columns (*p* < 0.05).

Figure 1A shows an image acquired using toluidine blue-staining, which verified that the target cell was a mesenchymal cell. Culture medium supplemented with melatonin exhibited a beneficial effect on cell proliferation; the results showed that the cell proliferation rate was significantly higher in the melatonin group (800, 2000 pg/mL) after 48–72 h culture (*p* < 0.05), and the proliferation rate was highest in the 800 ng/mL group after a 72-h culture (0.55 ± 0.06 vs. 0.38 ± 0.03, *p* < 0.01) (Figure 1B).

### 2.2. The Location and Expression of MT1 in Antler Mesenchymal Cells

**Figure 2:**
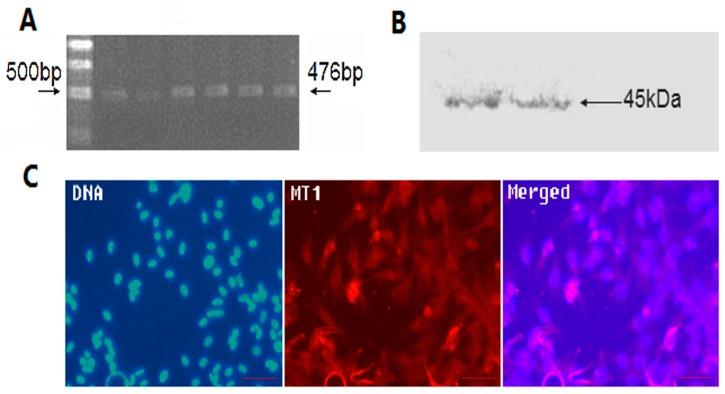
The expression of MT1 in mesenchymal cells. (**A**) Assayed using RT-PCR; objective strap size: 476 bp; (**B**) Assayed using Western blotting; objective strap size: 45 kDa; (**C**) Assayed using immunofluorescence; red indicates MT1 staining, blue indicated nucleus, stained with DAPI, Scale bar = 100 μm.

Data from RT-PCR, Western blotting, and immunofluorescent assay suggested that type I melatonin receptor (MT1) was present in the mesenchymal cells (Figure 2).

### 2.3. Construction of the Interference Fragment of MT1 Using Plasmid DNA

**Figure 3:**
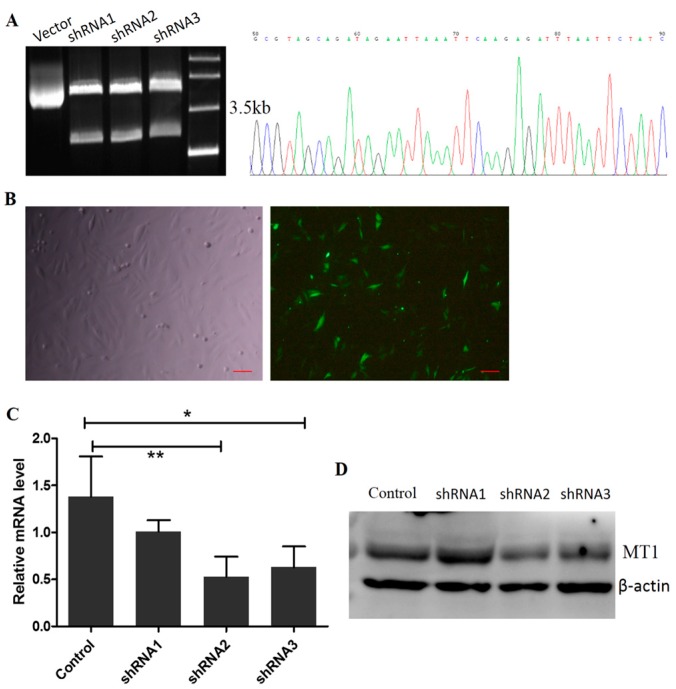
Efficiency identification of recombinant interference plasmids of MT1. (**A**) Enzyme digestion (**left**) and sequencing identification (**right**); (**B**) detection of transfection efficiency; (**left**) bright-field, (**right**) fluorescent light, Scale bar = 100 μm; (**C**) comparison of interference efficiency using qRT-PCR; (**D**) comparison of interference efficiency using Western blotting. * represents significant differences, *p* < 0.05, ** represents significant differences, *p* < 0.01.

Three fragments, shRNA1, shRNA2, and shRNA3 of the MT1 gene were connected with the linear plasmid, RNAi-Ready pSIREN-RetroQZsGreen. There were 4.5 KB and 2.1 KB bands with Hind III digestion, proving that recombinant plasmids were constructed successfully (Figure 3A(Left)) and the sequencing results showed no mutations in the inserted sequence (Figure 3A(Right)). The RNAi recombinant plasmids transfected and expressed normally in mesenchymal cells after 48 h (Figure 3B). qRT-PCR verified that the interference efficiencies of shRNA2/3 (0.53 ± 0.213, 0.63 ± 0.221 vs. 1.38 ± 0.43, respectively, *p* < 0.05) were significantly higher than that of shRNA1 (1.01 ± 0.121 vs. 1.38 ± 0.430) (Figure 3C). The results from Western blotting were consistent with those of qRT-PCR, and compared with the control, MT1 protein levels were decreased significantly in shRNA2/3 (*p* < 0.05). Thus, shRNA2 was selected for subsequent experiments (Figure 3D).

### 2.4. Effects of MLT/MT1 Knock-Down on the Viability of Mesenchymal Cells

**Figure 4:**
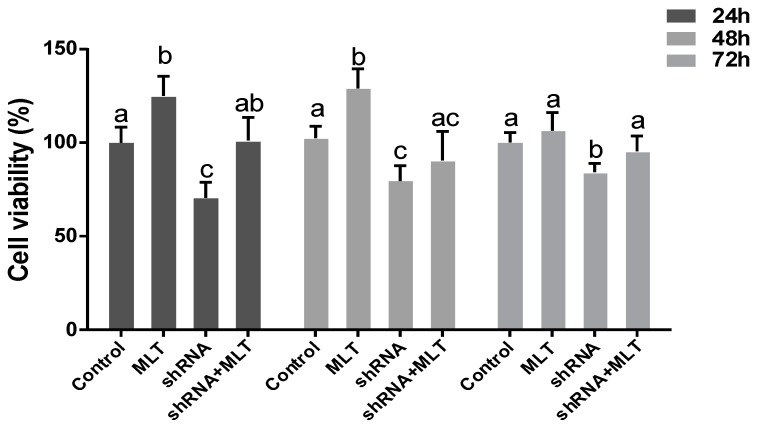
Effects of MLT/MT1 knock-down on the viability of mesenchymal cells. The different superscript letters (a–c) represent significant differences of these columns (*p* < 0.05).

Compared with the control group, melatonin significantly improved cell viability during cell culture ((24 h) 125 ± 10.6% vs. 100 ± 8.4%, (48 h) 129 ± 10.5% vs. 102 ± 6.3%, respectively, *p* < 0.05). Cell viability in the shRNA group was significantly lower than in the control ((24 h) 70 ± 7.4% vs. 100 ± 8.4%, *p* < 0.05, (48 h) 79 ± 8.2% vs. 102 ± 6.3%, *p* < 0.01, (72 h) 84 ± 4.8% vs. 100 ± 5.3%, *p* < 0.05). Melatonin partially relieved the abnormal phenotype caused by MT1-knock down, with respect to comparing the cell viability between the shRNA and shRNA + MLT groups (Figure 4).

### 2.5. Effects of MT1 Knock-Down on Cell Proliferation-Related Gene Expression

**Figure 5:**
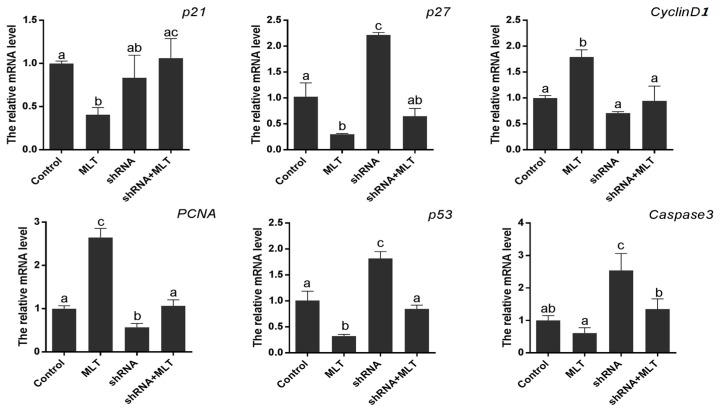
Effect of MT1 knock-down on genes expression. The different superscript letters (a–c) represent significant differences of these columns (*p* < 0.05).

The expression of cell proliferation-related genes was also detected. The results suggested that the expression of pro-proliferation genes (*PCNA*, *cyclinD1*) was significantly increased in the melatonin-treated group (2.65 ± 0.209 vs. 1.00 ± 0.039, 1.79 ± 0.079 vs. 1.00 ± 0.027, respectively. *p* < 0.01). Anti-proliferation genes, including *p21* and *p27*, were down-regulated by melatonin treatment compared to the control (0.41 ± 0.048 vs. 1.00 ± 0.016; 0.30 ± 0.009 vs. 1.03 ± 0.154, respectively. *p* < 0.01); MT1 knock-down (shRNA group) inhibited the expression of *PCNA* and *cyclinD1* significantly (0.57 ± 0.051 vs. 1.00 ± 0.039, *p* < 0.01, 0.71 ± 0.015 vs. 1.00 ± 0.027, *p* < 0.05), while it promoted the expression of *p27* (2.21 ± 0.033 vs. 1.00 ± 0.039, *p* < 0.01). Lastly, the changes of *PCNA*, *CyclinD1*, and *p27*, caused by RNAi, were all partially relieved by melatonin (shRNA + MLT). Apoptosis-related genes (*p53* and *Caspase3*) were also detected. Melatonin significantly decreased the expression of *p53* (0.32 ± 0.020 vs. 1.01 ± 0.103, *p* < 0.01), while MT1 knock-down significantly promoted *p53* and *Caspase3* expression (1.82 ± 0.077 vs. 1.01 ± 0.103, 2.54 ± 0.369 vs. 1.00 ± 0.100, respectively. *p* < 0.01). The changes in *p53* and *Caspase3* caused by RNAi were all partially relieved by melatonin (shRNA + MLT) (Figure 5).

### 2.6. Effects of MT1 Knock-Down on IGF/IGF1-R Signaling

**Figure 6:**
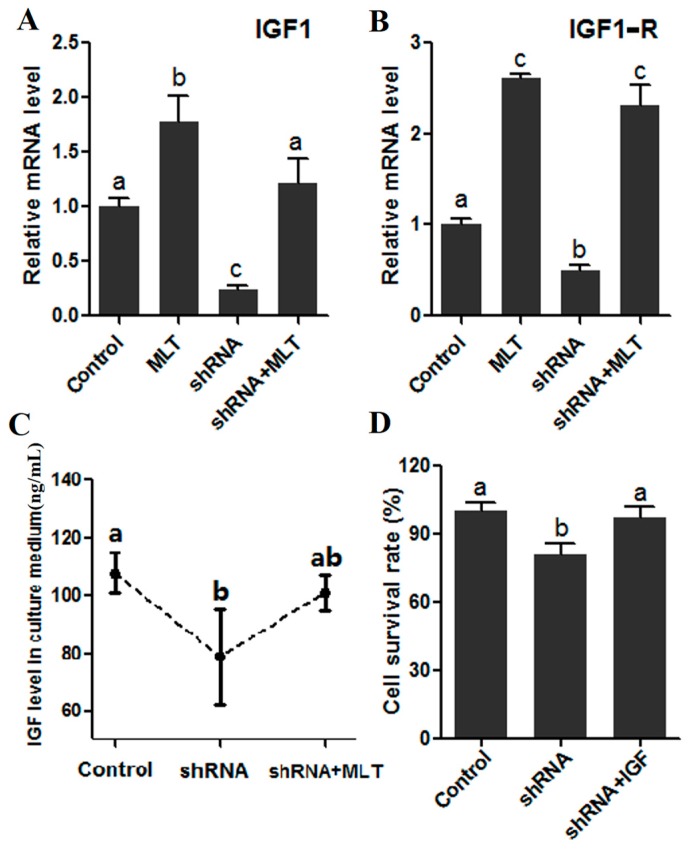
Effect of MT1 knock-down on IGF/IGF1-R signaling. (**A**) The expression of IGF1; (**B**) the expression of IGF1-R; (**C**) IGF level in culture medium; (**D**) the effect on the cell survival rate. Different superscript letters (a–c) represent significant differences of these columns (*p* < 0.05).

It was observed that melatonin significantly increased the expression of IGF1 and its trans-membrane receptor, IGF1R (1.77 ± 0.240 vs. 1.00 ± 0.010, 2.61 ± 0.040 vs. 1.00 ± 0.010, respectively, *p* < 0.01). Contrary to melatonin supplement, MT1 knock-down (shRNA group) significantly inhibited the expression of IGF1 and IGF1R (0.24 ± 0.040 vs. 1.00 ± 0.080, 0.49 ± 0.060 vs. 1.00 ± 0.020, respectively, *p* < 0.01), while the addition of melatonin in the shRNA-group relieved the expression tendency caused by RNAi (Figure 6A,B). Consistent with gene expression, the IGF-synthesis ability and cell survival rates were impaired in the shRNA group (78.67 ± 16.570 vs. 107.77 ± 7.060 ng/mL, *p* < 0.05, 0.81 ± 0.050 vs. 1.00 ± 0.040, respectively, *p* < 0.05), while melatonin supplementation also significantly abated the adverse tendency caused by RNAi (101.00 ± 6.220 vs. 107.77 ± 7.060 ng/mL, 0.97 ± 0.050 vs. 1.00 ± 0.040, respectively) (Figure 6C,D).

## 3. Discussion

Mesenchymal cells were located in the breeding area of antlers. During antler regeneration, the periosteal stem cells constantly differentiate to form mesenchymal cells, and then transform into cartilage cells, which promote antler growth [33,34]. Thus, the optimum metabolic conditions of mesenchymal cells sustained the rapid growth of the deer antler [3,35,36]. It is important to evaluate the regulatory mechanisms of mesenchymal cell proliferation and new measures improving antler production that may originate from them.

Melatonin, a critical hormone mainly secreted by the pineal gland, plays a key role in maintaining redox balance and biological rhythm control. It is believed that melatonin performs its functions via two pathways: Direct ROS (Reactive oxygen species)-scavenging action and a receptor-dependent (MT1, MT2) method [37,38,39]. An increasing number of studies have found that, except for the pineal gland, other organs also have the capability of melatonin synthesis, and constitute the paracrine/autocrine signaling loop in order to participate in the regulation of local metabolism [40,41]. For example, melatonin works as a local signal in the ovary to ameliorate mouse oocyte quality and participate in the regulation of luteinization [22,24]. The regeneration of antler is a periodic event. During antler regeneration, the concentration of melatonin in serum shows changes regularly, which suggests that melatonin may be involved in this process [42]. In the current research, we studied the effects of melatonin signals on mesenchymal cell proliferation with a cell model, and then analyzed the feasible mechanisms.

In this study, we first found that exogenous melatonin (400–2000 pg/mL) significantly promoted mesenchymal cell proliferation in vitro (Figure 1), which is consistent with previous reports that melatonin promotes granulosa cell and stem cell proliferation by ameliorating mitochondrial function, and also decreased ROS production and inhibited cell apoptosis [43,44,45]. However, the mechanisms by which melatonin promotes mesenchymal cell proliferation may be distinctive, as the MT1 receptor was detected in mesenchymal cells (Figure 2). Thus, we speculated that the action of melatonin in promoting mesenchymal proliferation was mediated by MT1. In order to verify this hypothesis, the optimal interference plasmid of MT1 was constructed and selected to knock down MT1 (Figure 3). Expectedly, MT1 knock-down significantly inhibited cell proliferation. Although melatonin supplementation rescued this negative action of MT1 knock-down, the beneficial effect of melatonin was crippled when compared with the melatonin-treated group (Figure 4). These data accurately confirmed that the MT1 receptor mediated the positive effects of melatonin on mesenchymal cell proliferation. Subsequently, the effects of MLT/MT1 signaling on proliferation-associated gene expression was studied; excessive activation of MLT/MT1 signaling using exogenous melatonin promoted the expression of pro-proliferation genes (*PCNA*, *cyclinD1*), and the expression of cyclin-dependent kinase inhibitor (*p21*, *p27*) and apoptosis-related genes (*p53*, *Caspase3*) were both down-regulated. The expression of these genes displayed the opposite pattern as MLT/MT1 attenuated using RNAi (Figure 5). These data suggested that the variation of proliferation-associated and apoptosis-related gene expression may contribute to increased proliferation of mesenchymal cells, stimulated by MLT/MT1 signaling.

The level of variation of insulin growth factor I (IGF1) was consistent with the growth and degradation of antler. The leading role of IGF1 in antler regeneration has been extensively validated, and it is believed that proliferation-related genes (*cyclinD1*, *PCNA*, *p21*, and *p27*, etc.) are the crucial target genes of IGF1/IGF1-R signaling, which contributes to antler regeneration [46,47]. Early studies indicated that a synergy may exist between IGF1 and melatonin signals [30]; nevertheless, thus far, no immediate evidence supports this. In this research, we investigated the relationship between MLT/MT1 and IGF1/IGF1-R during mesenchymal cell proliferation. Convincingly, the results suggested that MLT/MT1 signaling improved the expression of IGF1 and IGF1-R; meanwhile, MT1 knock-down significantly decreased the level of IGF1 (Figure 5A–C). Moreover, IGF rescued the negative effect of MT1 knock-down on mesenchymal cell proliferation (Figure 5D). These data adequately verified that IGF1/IGF1-R signaling worked as the downstream signaling of MLT/MT1 to regulate antler mesenchymal cell proliferation.

## 4. Materials and Methods

### 4.1. Sample Collection and Culture of Mesenchymal Cells

Deer antler samples were taken by the sika deer company of Jinsanxin (Wuhan, China). Interstitial tissue was cut up in 1× PBS, centrifuged at 800 rpm for 5 min and this was repeated three times; using 0.2% collagenase II (Sigma Aldrich, St. Louis, MO, USA) digestion for 30 min. Mesenchymal cells were obtained from antler of sika deer. Mesenchymal cells were grown in a culture dish in DMEM/High GLUCOSE (Hyclone, GE Healthcare, Issaquah, WA, USA) medium supplemented with 10% fetal bovine serum (FBS, Gibco BRL, Gaithersburg, MD, USA), 60 mg/mL penicillin, and 50 mg/mL streptomycin. All cultures were maintained at 37 °C in a humidified atmosphere of 5% CO_2_.

### 4.2. RNAi Plasmid Restructuring of MT1

RNAi-Ready pSIREN-RetroQZsGreen was stored by the College of Animal Science and Technology, Key Laboratory of Agricultural Animal Genetics, Breeding and Reproduction, Ministry of Education, Huazhong Agricultural University. The mRNA sequence of MT1 (ACCESSION: JN038179) was used for the target sequence of RNAi. Oligonucleotide sequences for the generation of siRNAs used in this study were designed using the Ambion siRNA Target Finder at the Ambion Inc. web (www.ambion.com/techlib/misc/siRNA_finder.html), sequence synthesis was conducted by Shanghai Sangon Limited (Shanghai, China). The three interference sequences were named shRNA1, shRNA2, and shRNA3 (Table 1). For transient transfection, mesenchymal cells were washed with 1× PBS, transfections were carried out using a Lipofectamine LTX kit (Invitrogen, Eugene, OR, USA) according to the manufacturer’s directions. Desired plasmids were diluted in Opti-MEM medium (Lifetechnology, Waltham, MA, USA), incubated for 5 min and later mixed with LTX for 30 min. Mesenchymal cells were collected for protein extraction or other analyses after transfection.

### 4.3. Western Blot Assay

Plasmid transfection was washed rapidly with ice-cold 1× PBS after 72 h, and later placed into a centrifugation tube containing 100 μL of ice-cold RIPA buffer, for 30 min. Then, lysates were centrifuged at 12,000 rpm for 5 min, and the supernatant was obtained and stored at −20 °C. Total protein was separated on 10% polyacrylamide gel, and then transferred to a 0.45-μm PVDF membrane. The membrane was placed in 5% nonfat dry milk in Tris Buffered Saline Tween (1× TBST) at room temperature for 1 h, and then incubated with goat polyclone anti-MT1 (Santa Cruz, CA, USA, 1:2000 for WB and 1:100 for IF), antibody or mouse monoclonal anti-β-actin antibody (1:5000, Biodragon Immunotechnologies, Beijing, China) diluted in blocking buffer at 4 °C overnight. After incubation with the primary antibodies, membranes were washed three times with TBST solution, and then incubated with HRP-conjugated rabbit anti-goat IgG antibody (1:2000, Boster Biological Technology, Co. Ltd., Beijing, China) or HRP-conjugated goat anti-mouse IgG antibody at room temperature for 2 h. After washing three times with TBST solution, the membranes were incubated with the ECL chemiluminescence reagent for 5 min, and later exposed to X-ray film for the observation of protein bands. Band intensities were measured with Gel-Pro analyzer 4.0 (Media Cybernetics, Rockville, MD, USA).

### 4.4. Cell Proliferation Test

The experiment was divided into a control group and experimental groups (added with 400, 800, 1200, 1600, and 2000 pg/mL melatonin). Plasmid transfection was performed after 24 h, 48 h, and 72 h; the cell proliferation rate was detected by MTT (Dojindo, Kyushu, Japan). Each group added with 50 μL MTT and cultured for 4 h, then 150 μL DMSO (Sigma Aldrich, St. Louis, MO, USA) was added in each group and incubated for 10 min in a shaking bed at 37 °C, using enzyme standard instruments to detect the λ = 550 nm absorbance values.

### 4.5. Immunofluorescence Assay

For the detection of the sub-cellular localization pattern of MT1, indirect immunofluorescence staining was conducted after transfection. Cells were cultured in a confluent monolayer on glass cover slips and transfected with plasmids. At 48 h after transfection, cells were washed three times with PBS and fixed in 4% paraformaldehyde for 30 min. After washing three times with PBS, cells were permeabilized for 20 min with 0.5% Triton-100 at room temperature, and then blocked in 5% bovine serum albumin. Then, the cells were incubated with anti-MT1 primary antibody overnight at 4 °C. The next day, cells were washed three times with PBS, and incubated with FITC-conjugated secondary antibody (1:100, Boster Biological Technology, Beijing, China) in the dark for 1 h. The nuclei of the cells were stained with 10 μg/mL 4,6-diamidino-2-phenylindole (DAPI, Servicebio, Wuhan, China) for 5 min. After that, a cover slip was mounted and analyzed under a Nikon TE2000-U Fluorescence microscope (Nikon, Tokyo, Japan).

### 4.6. Real-Time PCR Analysis

Real-time quantitative PCRs were run using a QuantiFast SYBR Green PCR Kit on a BIORAD CFX Manager Machine (Bio-Rad, Hercules, CA, USA). The *MT1,* *IGF1,* *IGF1-R*, *Cyclin D1*, *PCNA*, *P21*, *P27* and *P53*, as well as the β-actin primers (shown in Table 2) were designed using Primer Premier 5 software and synthesized (TSINGKE Biological Technology Company, Wuhan, China); samples were prepared according to the manufacturer’s instructions. The total reaction volume of 10 μL contained 5 μL Master Mix, 1 μL primers, 1 μL diluted cDNA and 3 μL ddH_2_O. The samples were then centrifuged at 1000× *g* at 4 °C for 1 min. All cDNA samples were amplified under the following conditions: 95 °C for 1 min, 40 cycles at 95 °C for 10 s, and 55 °C for 30 s. The data were subsequently quantified according to the comparative cycle threshold method, and expression levels were presented as 2^−ΔCT^ normalized to the β-actin housekeeping gene.

### 4.7. Enzyme-Linked Immunosorbent Assay (ELISA) Assay

IGF1 was detected using an indirect ELISA method. The 96-well immunoplates were coated with 100 ng of IGF1 (PeproTech, Rocky Hill, NJ, USA) diluted in bicarbonate buffer (pH 9.6) overnight at 4 °C. Following blocking with 5% (*w*/*v*) dry skimmed milk in PBS (pH 7.2) for 1 h at 37 °C, 1:50 dilution of plasma samples in 0.05% Tween-20 in PBS (PBST) were added to the wells and the plates were incubated for 1 h at 37 °C. Each sample was tested in triplicate; positive and negative controls were also used. Bound antibody was detected by the addition of 100 μL of HRP-labeled goat anti-rat IgG antibodies in PBST and incubated for 1 h at 37 °C. To develop the ELISA results, 10 mg of tetramethylbenzidine (TMB tablets, Sigma-Aldrich, St. Louis, MO, USA) were dissolved in absolute ethanol that was mixed with phosphate–citrate buffer (pH 5.0) using H_2_O_2_ as a substrate, and then incubated for 10 min at 37 °C. Reactions were terminated with 2 mol/L H_2_SO_4_ and the resulting optical density (OD) was measured at 450 nm in a plate reader (Thermo Electron Corporation, PerkinElmer, Waltham, MA, USA).

### 4.8. Data Analysis and Statistics

All experiments were repeated at least three times independently. Bio-Rad IQ5 software (Bio-Rad Laboratories, Hercules, CA, USA) and Microsoft Office Excel were used to analyze RT-PCR, using the 2^−ΔΔCT^ method to calculate mRNA expression. The data were represented as mean ± SEM and analyzed using SPSS 19.0 software (SPSS, Chicago, IL, USA). If differences were found after One-Way ANOVA, significance was measured by using Tukey’s post hoc test. A value of *p* < 0.05 was considered significantly different and extremely significant when *p* < 0.01.

## 5. Conclusions

In conclusion, the current experiments suggested that melatonin regulates antler mesenchymal cell proliferation via the MT1 receptor. Mechanism research verified that MLT/MT1 signaling enhanced IGF1/IGF1-R signaling by increasing both IGF1 and IGF1-R expression. This study supplies definite proof, using a cell model, to support the view that melatonin promotes antler regeneration by activating IGF1/IGF1-R. This study provides an important theoretical basis to improve antler production using melatonin.

## Figures and Tables

**Table 1:** shRNA sequence of MT1.

Name	Sense/Antisense	Sequence Sense
shRNA1	Sense	5′-GATCCGGTCAATAGAGAATTAACAGTTCAAGAG ACTGTTAATTCTCTATTGACCTTTTTTAAGCTTG-3′
	Antisense	5′-AATTCAAGCTTAAAAAAGGTCAATAGAGAATTA ACAGTCTCTTGAACTGTTAATTCTCTATTGACCG-3′
shRNA2	Sense	5′-GATCCGCGTAGCAGATAGAATTAAATTCAAGAGA TTTAATTCTATCTGCTACGCTTTTTTAAGCTTG-3′
	Antisense	5′-AATTCAAGCTTAAAAAAGCGTAGCAGATAGAAT TAAATCTCTTGAATTTAATTCTATCTGCTACGCG-3′
shRNA3	Sense	5′-GATCCGGGATCTATTCCTGCACCTTCATTCAAGAGA TGAAGGTGCAGGAATAGATCCCTTTTTTAAGCTTG-3′
	Antisense	5′-AATTCAAGCTTAAAAAAGGGATCTATTCCTGCACC TTCATCTCTTGAATGAAGGTGCAGGAATAGATCCCG-3′

**Table 2:** Primers used in this study for real-time PCR.

Gene	Primer Sequence (5′-3′)	Tm (°C)	Product Size (bp)
*β-actin*	F: TGACCCTTAAGTACCCCATCGA	60	85 bp
	R: TTGTAGAAGGTGTGGTGCCAGAT		
*MT1*	F: TGGCTGTTTGTGGCCAGTTA	60	158 bp
	R: ACGTGATTGGAGCTATCCGC		
*IGF1*	F: TGTGATTTCTTGAAGCAGGTGA	60	96 bp
	R: CGTGGCAGAGCTGGTGAAG		
*IGF-1R*	F: GCACCATCTTCAAAGGCAATC	60	95 bp
	R: GAGACCAAGGCGTGGGAGT		
*Cyclin D1*	F: GCGCAGACCTTCGTTGCCCT	60	123 bp
	R: GCCGTTGGCGCTTCCCAGAT		
*PCNA*	F: CCTTGGTGCAGCTAACCCTT	60	94 bp
	R: TTGGACATGCTGGTGAGGTT		
*P21*	F: GACCACTTGGACCTGTCGCT	60	183 bp
	R: GGGTTAGGGCTTCCTCTTGG		
*P27*	F: AGTGTCTAACGGGAGTCCGA	60	213 bp
	R: CACTCGTACTTGCCCTCCAG		
*P53*	F: GAAGACCTACCCTGGCAATTAC	60	103 bp
	R: AGAACAGCTTGTTAAGGGAAGG		
